# The impact of a second MRI and re-biopsy in patients with initial negative mpMRI-targeted and systematic biopsy for PIRADS ≥ 3 lesions

**DOI:** 10.1007/s00345-023-04578-7

**Published:** 2023-09-27

**Authors:** Fabio Zattoni, Leonor J. Paulino Pereira, Giancarlo Marra, Massimo Valerio, Jonathan Olivier, Ignacio Puche-Sanz, Pawel Rajwa, Martina Maggi, Riccardo Campi, Daniele Amparore, Sabrina De Cillis, Zhuang Junlong, Hongqian Guo, Giulia La Bombarda, Andrea Fuschi, Alessandro Veccia, Francesco Ditonno, Alessandro Marquis, Francesco Barletta, Riccardo Leni, Sergio Serni, Veeru Kasivisvanathan, Alessandro Antonelli, Fabrizio Dal Moro, Juan Gomez Rivas, Roderick C. N. van den Bergh, Alberto Briganti, Giorgio Gandaglia, Giacomo Novara

**Affiliations:** 1 Urologic Unit, Department of Surgery, Oncology and Gastroenterology, University of Padova, Padua, Italy; 2 Department of Urology, Sint Antonius Hospital, Utrecht-Nieuwegein, The Netherlands; 3 Division of Urology, Department of Surgical Sciences, Molinette Hospital, University of Turin, 10126 Turin, Italy; 4 Department of Urology, Geneva University Hospital, University of Geneva, Geneva, Switzerland; 5 Department of Urology, Lille University, Lille, France; 6 Department of Urology, Instituto de Investigación Biosanitaria Ibs.Granada, Hospital Universitario Virgen de Las Nieves (HUVN), Granada, Spain; 7 Department of Urology, Medical University of Vienna, Vienna, Austria; 8 Department of Maternal-Infant and Urological Sciences, Sapienza University of Rome, Rome, Italy; 9 Unit of Urological Robotic Surgery and Renal Transplantation, Careggi Hospital, University of Florence, Florence, Italy; 10 School of Medicine, Division of Urology, Department of Oncology, San Luigi Gonzaga Hospital, Orbassano, Italy; 11 Institute of Urology, Nanjing Drum Tower Hospital, Medical School of Nanjing University, Nanjing University, Nanjing, Jiangsu People’s Republic of China; 12 Department of Urology, Azienda Ospedaliera Universitaria Integrata Verona, Verona, Italy; 13 Division of Oncology/Unit of Urology, URI, IRCCS Ospedale San Raffaele, Milan, Italy; 14 Division of Surgery and Interventional Science, UCL, London, UK; 15 Department of Urology, Hospital Clínico San Carlos, Madrid, Spain; 16 Faculty of Pharmacy and Medicine, Sapienza University of Rome, Latina, Italy; 17 Urology Unit, Department of Medico, Surgical Sciences and Biotechnologies, Sapienza University of Rome, Rome, Italy; 18 Department of Experimental and Clinical Medicine, University of Florence, Florence, Italy; 19 Department of Urology, Nanjing University, Nanjing, China; 20 Department of Urology, Medical University of Silesia, Zabrze, Poland; 21 School of Medicine, Division of Urology, Department of Oncology, University of Turin, Turin, Italy

**Keywords:** Prostate cancer, Diagnosis prostate MRI, Negative biopsy, Target prostate biopsy, Targeted biopsy

## Abstract

**Objective:**

To evaluate the proportions of detected prostate cancer (PCa) and clinically significant PCa (csPCa), as well as identify clinical predictors of PCa, in patients with PI-RADS >  = 3 lesion at mpMRI and initial negative targeted and systematic biopsy (initial biopsy) who underwent a second MRI and a re-biopsy.

**Methods:**

A total of 290 patients from 10 tertiary referral centers were included. The primary outcome measures were the presence of PCa and csPCa at re-biopsy. Logistic regression analyses were performed to evaluate predictors of PCa and csPCa, adjusting for relevant covariates.

**Results:**

Forty-two percentage of patients exhibited the presence of a new lesion. Furthermore, at the second MRI, patients showed stable, upgrading, and downgrading PI-RADS lesions in 42%, 39%, and 19%, respectively. The interval from the initial to repeated mpMRI and from the initial to repeated biopsy was 16 mo (IQR 12–20) and 18 mo (IQR 12–21), respectively. One hundred and eight patients (37.2%) were diagnosed with PCa and 74 (25.5%) with csPCa at re-biopsy. The presence of ASAP on the initial biopsy strongly predicted the presence of PCa and csPCa at re-biopsy. Furthermore, PI-RADS scores at the first and second MRI and a higher number of systematic biopsy cores at first and second biopsy were independent predictors of the presence of PCa and csPCa. Selection bias cannot be ruled out.

**Conclusions:**

Persistent PI-RADS ≥ 3 at the second MRI is suggestive of the presence of a not negligible proportion of csPca. These findings contribute to the refinement of risk stratification for men with initial negative MRI-TBx.

**Supplementary Information:**

The online version contains supplementary material available at 10.1007/s00345-023-04578-7.

## Introduction

Prostate cancer (PCa) diagnosis has undergone a significant transformation with the introduction of multiparametric MRI (mpMRI) in recent years. Using mpMRI before a prostate biopsy has revolutionized risk stratification in various clinical scenarios. By using MRI-targeted biopsy (MRI-TBx) for suspicious lesions, the incidental detection of clinically insignificant PCa has been reduced [1], and the diagnosis of the insignificant disease has been minimized [2, 3]. However, there is ongoing controversy regarding whether MRI-targeted biopsy can result in a grade shift through overgrading the index lesion, potentially leading to unnecessary overtreatment of PCa that could otherwise be appropriately managed through active surveillance [4]. Furthermore, a negative re-biopsy mpMRI has demonstrated an overall negative predictive value (NPV) of 82% for all cancers and 98% for ISUP ≥ 2 cancer [5]. Despite the growing consensus among urologists regarding the routine use of mpMRI before prostate biopsy, the specificity remains limited, reaching only 37% [6]. Interestingly, limited data are available for patients who have undergone negative MRI-TBx and systematic prostate biopsy (RBx) following an initial positive mpMRI. A recent mini-systematic review focused on the proportion of PCa detected in the setting of repeated biopsy [7]. The review reported an overall cancer detection rate ranging from 0% to 7.5% and a clinically significant Pca (csPCa) detection rate ranging from 0% to 2.5% for patients with a PI-RADS 3 lesion. Notably, patients with a Likert 5 lesion had an overall cancer detection rate of 87.5%. However, this systematic review had limitations, as it relied on a small number of studies with a limited patient population. In particular, little is known regarding the evolution of MRI lesions that were negative during the initial MRI-TBx and their status upon a second MRI.

In response to the European Association of Urology’s expressed need for its guideline update, we aim to address this knowledge gap and evaluate the proportions of detected Pca and csPCa, as well as identify clinical predictors of Pca, in patients with initial negative MRI-TBx and RBx who underwent a second mpMRI and a repeated biopsy.

## Materials & methods

Internal Review Board approval was obtained for the present study and retrospective data collection in accordance with the policies of each participating institution.

**Fig. 1:**
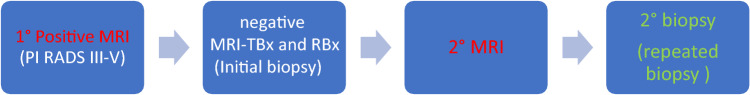
Path from first positive MRI to second biopsy following Subsequent MRI

A total of 290 patients who met the following criteria were included from 10 tertiary referral centers: positive MRI (PI-RADS ≥ 3) with negative MRI-TBx and RBx (initial biopsy). Patients were included if they had a positive MRI (PI-RADS ≥ 3) along with negative results on both MRI-TBx and RBx (initial biopsy). All patients had a second prostate mpMRI and a subsequent biopsy (re-biopsy), including either MRI-TBx and/or RBx (Fig. 1).

In the second MRI, upgrading and downgrading of PI-RADS were defined as any increase or decrease in the PI-RADS value compared to the first MRI, respectively.

The exclusion criteria were patients who underwent a systematic biopsy before the initial MRI-TBx and RBx, and patients in follow-up with MRIs only after the initial biopsy.

### Prostate biopsy techniques

A multiparametric MRI was performed before the biopsy, following each institution’s protocol. All centers utilized the PI-RADSv2 scoring system to assess MRI findings [8]. Expert genitourinary radiologists reviewed all MRIs in accordance with the ESUR/ESUI consensus for image acquisition, interpretation, and radiologists’ training [9]. Transrectal or transperineal targeted biopsies were performed by experienced urologists with more than 100 cases using their preferred biopsy approach [10]. Targeted biopsies were performed using dedicated biopsy fusion software or cognitive methods, according to the expertise of each center. Transperineal TBx was performed with a brachytherapy grid or freehand technique under general or local anesthesia.

### Statistical analysis

Categorical variables were presented as frequencies, while continuous variables were reported as mean ± standard deviation (SD) for normally distributed variables and as median and interquartile range (IQR) for non-normally distributed variables. Differences between categorical and continuous variables were assessed using either Chi-square, *T* test, or Mann–Whitney *U* test, as appropriate. The differences between continuous matched variables were assessed with the Wilcoxon sign-rank test. Univariable (UVA) and multivariable (MVA) logistic regression analyses were performed to evaluate predictors of PCa and csPCa at the moment of re-biopsy. CSPca was defined as any ISUP ≥ 2 cancer. Two models were created: one using available clinical and radiological information immediately after the initial biopsy (model A: age, PSA at initial biopsy, prostate volume at initial biopsy, PSA Density, PI-RADS score at first mpMRI, cT stage at first mpMRI, route for first biopsy, registration mode for MRI-TBx at first biopsy, number of RBx cores at first biopsy, histology of first biopsy) and the second using available information at the time of re-biopsy (model B: PSA at re-biopsy, prostate volume at second mpMRI, PSA Density, PI-RADS score at second mpMRI, evolution of the initial mpMRI lesion, presence of new MRI lesions, cT stage at second mpMRI, route for re-biopsy, registration mode for MRI-TBx at re-biopsy, number of RBx cores at re-biopsy, histology of first biopsy). Covariates included in the model were selected based on univariable results with *p* values ≤ 0.1. Variables with suspicious interaction terms were adjusted accordingly.

An intraclass correlation coefficient (ICC) with 95% confidence interval was calculated to compare the concordance within the same patients of the first and second MRI, second MRI with the worst ISUP at re-biopsy. Similarly, ICC was calculated to assess the concordance of ISUP scores between MRI-TBx, and the combination of MRI-TBx and RBx with the final pathology for patients treated with RP. A significance level of *p* < 0.05 was used for all tests. Statistical analyses were performed using SPSS version 23 (IBM, Armonk, NY, USA).

## Results

Table 1 describes the characteristics at the moment of the initial and repeated biopsy for the 290 evaluated patients. The interval from the initial to repeated mpMRI and from the initial to repeated biopsy was 16 mo (IQR 12–20) and 18 mo (IQR 12–21), respectively.

**Table 1:** Characteristics of the 290 patients evaluated at the initial biopsy

Characteristics	
At the moment of initial biopsy	
Median Age (IQR)	65 (61–71)
Therapy with 5-ARI	103 (50)
Median PSA (IQR)	6.5 (4.7–9.0)
Median prostate volume (IQR)	53 (40–67)
PSA density (IQR)	0.12 (0.09–0.19)
Positive DRE	107 (37%)
Clinical stage cT2 at initial MRI	229 (79%)
Max diameter lesion (mm) at initial MRI	9 (6–12)
PI-RADS at initial MRI	
3	137 (47%)
> 3	153 (53%)
Biopsy type	
Cognitive	94 (32%)
Software fusion	196 (68%)
Biopsy route	
Transrectal	137 (47%)
Transperineal	153 (53%)
Median number of systematic biopsies (IQR)	12 (11–13)
Median number of targeted biopsies (IQR)	4 (2–5)
ASAP histology at initial biopsy at initial biopsy	59 (20)
At the moment of repeated biopsy	
PSA at biopsy (IQR)	8.3 (5.6–11.3)
Prostate volume (IQR)	60 (45–75)
PSA density (IQR)	0.14 (0.1–0.22)
Clinical stage cT2 at second MRI	195 (70%)
Max diameter lesion (mm) at second MRI	10 (8–14)
PI-RADS at second MRI	
< 3/ negative	25 (8.7%)
3	91 (31%)
4	137 (47%)
5	37 (13%)
Biopsy type	
Systematic	28 (10%)
Cognitive	46 (16%)
Software fusion	216 (74%)
Biopsy route	
Transrectal	118 (40%)
Transperineal	59 (60%)
Median number of systematic biopsies	12 (10–13)
Median number of targeted biopsies	4 (2–5)

From initial to repeated biopsy, there has been a significant increase in PSA (6.5 vs. 8.3), prostate volume (53 cc vs. 60 cc), PSAD (0.13 vs. 0.14 ng/ml/cc), and max diameter of the lesion (9 mm vs. 10 mm) (all *p* values < 0.01). Fusion software biopsies were performed in 68% of the initial biopsy and 74% of the repeated biopsy. The transperineal biopsy route was chosen in 53% of the initial biopsy and 60% of the repeated biopsy.

Table 2 provides insights into the follow-up of prostate lesions described at the first MRI. Notably, 42% of the patients showed the presence of new lesions at the second MRI. Among the lesions described at the first MRI with a PI-RADS classification, 42% remained stable, while 39% exhibited upgrading and 19% showed downgrading. Among 131 men with PI-RADS 4 or 5 lesions at the initial MRI 20.1% of lesions were downgraded to PI-RADS ≤ 3 in the second MRI.

**Table 2:** Follow–up of prostate lesions described at first MRI

Presence of new lesions at second MRI	122 (42%)
Lesion described at first MRI	
Stable	121 (42%)
Upgrading	114 (39%)
Downgrading	55 (19%)
ISUP systematic biopsies at repeated biopsy	
Negative	200 (69%)
1	24 (8.3%)
2	27 (9.3%)
3	18 (6.2%)
4	6 (2.1%)
5	4 (1.4%)
Not performed	11 (3.8%)
ISUP at targeted biopsy at repeated biopsy	
Negative	170 (58.6%)
1	26 (9.0%)
2	33 (11.4%)
3	15 (5.2%)
4	2 (0.7%)
5	1 (0.3%)
Not performed	43 (14.8%)
ISUP target + systematic at repeated biopsy	
NEGATIVE	182 (62.0%)
1	34 (11.7%)
2	37 (12.8%)
3	27 (9.2%)
4	6 (2.1%)
5	4 (1.4%)
Pca diagnosis at repeated biopsy	108 (37.2%)
Pca diagnosis at subsequent biopsies	2 (0.7%)
Status at follow-up	
Negative	180 (62%)
No csPca	24 (8.3%)
csPca	86 (29.7%)
Interval from the initial to repeated mpMRI	16 mo (IQR 12–20)
Interval from the initial to repeated biopsy	18 mo (IQR 12–21)
Follow-up from second negative biopsy	20 months (5.7–34.7)
Therapy	
AS/WW	23/110 (20%)
Surgery	72/110 (65%)
RT	10/110 (10%)
Focal therapy	1/110 (1%)
Other	4/110 (4%)
T stage at RP	
pT2	34 (45%)
pT3a	30 (41%)
pT3b	10 (14%)
N stage at RP	
N0	40 (53%)
N1	7 (10%)
Nx	27 (37%)

Supplementary Table 1 presents the clinical and radiological triggers for repeat biopsy.

On the whole, 108 patients (37.2%) were diagnosed with PCa and 74 (25.5%) with csPCa at re-biopsy. Most RBx were negative (69%), with a smaller proportion falling into csPCa. MRI-TBx were negative in 58.6%. Supplementary table 2 provides detailed information regarding the location of newly diagnosed cancer, indicating whether it was found in the original MRI area of concern, new suspicious MRI areas, or through a systematic approach.

The majority of patients in both groups had negative biopsy results. Approximately 10% of patients in each group showed positive findings in the Target biopsy and Positive Pca at Systematic biopsy. Notably, the combined Positive Systematic + Target biopsy approach yielded higher detection rates, with 16.1% and 17.2% positivity for patients with Lesions detected at the first MRI and New lesions detected at the second MRI, respectively.

**Fig. 2:**
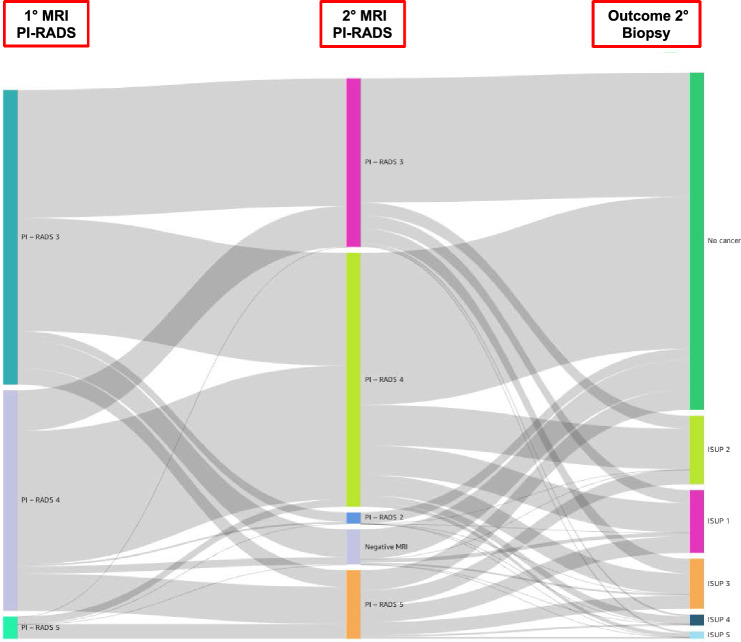
Sankey diagram on how patients move from initial PI-RADS to PI-RADS at second MRI and eventual diagnosis with second prostate biopsy

Figure 2 illustrates in a Sankey diagram the evolution of MRI lesions from the first to the second MRI and the subsequent diagnosis through prostate biopsy.

The median follow-up from second negative biopsy was 20 months (5.7–34.7). Further MRI and subsequent biopsy were performed in 19 patients, of whom only 2 patients were diagnosed with PC.

### Predictors of PCA and CS PCA

Tables 3 and 4 summarize UVA and MVA logistic regression analyses assessing predictors of PCa and csPCa, respectively. Models A include clinical and radiological information available at the moment of the initial negative biopsy, whereas models B include clinical and radiological information available at the moment of repeated biopsy.

**Table 3:** Univariable and multivariable logistic regression analyses predicting all PCa (model A with clinical and radiological information available at the moment of the initial negative biopsy and model B with clinical and radiological information available at the moment of repeated biopsy)

Covariates	Univariable analysis			Multivariable analysis		
	OR	95% CI of OR	*P* values	OR	95% CI of OR	*P* values
Model A (clinical and radiological information available at the moment of the initial negative biopsy)						
Age (continuous)	1.0	0.9–1.1	0.4	–	–	–
PSA at initial biopsy (continuous)	0.9	0.8–0.9	< 0.01	0.9	0.9–1.0	0.3
Prostate volume (ml) at initial biopsy (continuous)	0.9	0.9–0.9	< 0.01	0.9	0.9–0.9	0.02
PSA Density (continuous)	0.9	0.1–5.5	0.9	–	–	–
PI-RADS 3 versus 4–5	2.8	1.7–4.6	< 0.01	2.7	1.6–4.7	< 0.01
cT2 versus cT ≥ 3	1.0	0.6–1.9	0.8	–	–	–
Transrectal versus transperineal route	0.8	0.5–1.3	0.5	–	–	–
Cognitive versus software fusion biopsy	1.2	0.8–2.1	0.3	–	–	–
No. of target biopsy cores (**≤ **3 vs. > 3) at first biopsy	2.0	1.3–3.3	< 0.01	1.5	0.8–2.8	0.2
No. of systematic biopsy cores at first biopsy			< 0.01			0.03
No. systematic biopsy versus ≤ 12	0.8	0.4–1.8		0.3	0.3–1.7	0.3
No. systematic biopsy versus > 12	0.2	0.2–0.6		1.4	1.0–3.5	0.04
ASAP (presence vs. absence)	3.6	2.0–6.6	< 0.01	2.9	1.5–6.0	< 0.01
Model B (clinical and radiological information available at the moment of repeated biopsy)						
PSA at second MRI (continuous)	1.0	0.9–1.0	0.6	–	–	–
Prostate volume (ml) at second MRI (continuous)	1.0	0.9–1.0	0.08	0.9	0.9–1.0	0.07
PSA density at second MRI (continuous)	1.2	0.3–4.4	0.8	–	–	–
PI-RADS ≤ 3 versus 4–5 at second MRI	2.8	1.7–4.7	< 0.01	2.0 4.7	0.9–4.4 1.8–11.9	< 0.01* 0.05 < 0.01
cT at second MRI			< 0.01			
cT = 2 versus No cancer	4.9	1.1–22.1	0.03			
cT ≥ 3 versus No cancer	9.0	1.9–42.2	< 0.01			
Lesion described at first MRI with second MRI			0.02			0.9
Stable versus upgrading	1.8	1.0–3.0	0.03	1.1	0.6–2.2	0.7
Stable versus downgrading	0.8	0.4–1.6	0.4	1.0	0.5–2.3	0.9
Presence of new lesions at second MRI (yes vs. no)	1.1	0.7–1.8	0.6	–	–	–
Transrectal versus transperineal route at re-biopsy	1.2	0.7–1.9	0.5	–	–	–
Cognitive versus software fusion at re-biopsy			0.03			0.9
Systematic versus cognitive	3.6	1.3–10.0	0.02	1.2	0.3–4.4	0.8
Cognitive versus MRI target	1.7	0.7–4.1	0.2	1.2	0.4–4.1	0.7
N. of target biopsy cores at re-biopsy			0.2			
No fusion vs ≤ 3	0.9	0.4–2.1	0.9	–	–	–
No fusion versus > 3	1.5	0.7–3.3	0.2			
N. of systematic biopsy cores at re-biopsy			< 0.01			< 0.01
No systematic biopsy versus ≤ 12	0.6	0.2–0.9	0.03	0.5	0.2–1.0	0.06
No systematic biopsy versus > 12	2.2	1.0–4.6	0.047	2.1	0.9–5.1	0.09

*Interaction terms

**Table 4:** Univariable and multivariable logistic regression analyses predicting csPCa (model A with clinical and radiological information available at the moment of the initial negative biopsy and model B with clinical and radiological information available at the moment of repeated biopsy)

Covariates	Univariable analysis			Multivariable analysis		
	OR	95% CI of OR	*P* values	OR	95% CI of ORs	*P* values
Model A (clinical and radiological information available at the moment of the initial negative biopsy)						
Age (continuous)	1.0	0.9–1.1	0.5	–	–	–
PSA at biopsy A (continuous)	0.9	0.9–1.0	0.02	0.9	0.9–1.0	0.2
Prostate volume (ml) at biopsy A (continuous)	0.9	0.9–0.9	< 0.01	–	–	–
PSA Density (continuous)	0.2	0.02–2.0	0.2	–	–	–
PI-RADS (3 vs. > 3)	2.1	1.3–3.5	< 0.01	2.1	1.2–3.7	0.01
cT** ≥ **3 versus cT2	1.4	0.8–2.6	0.2	–	–	–
Transrectal versus Transperineal route	1.3	0.8–2.2	0.2	–	–	–
Cognitive versus software fusion biopsy at first biopsy	1.8	1.0–3.3	0.03	2.2	1.1–4.4	0.03
N. of target biopsy cores (**≤ **3 vs > 3) at first biopsy	2.7	1.6–4.6	< 0.01	1.5	0.8–3.0	0.2
N. of Systematic Biopsy cores at first biopsy			< 0.01			< 0.01
N. systematic biopsy versus ≤ 12	0.4	0.2–0.8	0.02	0.7	0.3–1.7	0.5
N. systematic biopsy versus > 12	1.5	0.7–3.3	0.3	2.4	0.9–6.0	0.06
ASAP (presence vs. absent)	3.2	1.8–5.8	< 0.01	2.3	1.1–5.0	0.02
Model B (clinical and radiological information available at the moment of repeated biopsy)						
PSA at second MRI (continuous)	1.0	0.9–1.0	0.9	–	–	–
Prostate volume (ml) at second MRI (continuous)	0.9	0.9–0.9	0.04	0.9	0.9–0.9	0.01
PSA density at second MRI (continuous)	1.6	0.4–6.0	0.4	–	–	–
PI_RADS (3 vs. 4–5) at second	2.7	1.5–4.7	< 0.01			< 0.01*
MRI				1.5 5.2	0.6–3.4 1.8–14	0.4 < 0.01
cT at second MRI			< 0.01			
cT = 2 versus no cancer	6.5	0.8–50.2	0.07			
cT** ≥ **3 versus no cancer	15.9	2.0–26.4	< 0.01			
Lesion described at first MRI vs second MRI			0.01			0.5
Stable versus upgrading	2.2	1.3–4.0	< 0.01	1.4	0.7–3.1	0.3
Stable versus downgrading	1.1	0.5–2.4	0.7	1.6	0.6–3.9	0.3
Presence of new lesions at second MRI	1.0	0.5–1.6	0.9	–	–	–
Transrectal versus transperineal route at re-biopsy	1.3	0.8–2.2	0.3	–	–	–
Cognitive versus software fusion at re-biopsy			0.07			0.2
Systematic versus cognitive	4.9	1.3–18.6	0.02	3.9	0.6–26	0.2
Cognitive versus MRI target	3.7	1.0–12.6	0.04	5.8	0.9–38	0.07
N. of target biopsy cores at re-biopsy			0.01			0.2
No fusion versus ≤ 3	0.9	0.3–2.2	0.8	0.7	0.2–2.3	0.5
No fusion versus > 3	2.0	0.9–4.6	0.09	1.4	0.4–5.2	0.6
N. of systematic biopsy cores at re-biopsy			< 0.01			< 0.01
No systematic biopsy versus ≤ 12	0.4	0.2–0.7	< 0.01	0.5	0.2–1.3	0.1
No systematic biopsy versus > 12	1.9	0.9–4.1	0.09	3.3	1.3–8.6	0.01

*Interaction terms

At MVA, the presence of a higher PI-RADS score, the presence of ASAP at first biopsy, and increasing number of systematic biopsy cores were independent predictors of any PCA and csPCa in models A. A higher PI-RADS score, cT stage at the second MRI, and the higher number of systematic biopsy cores at repeated biopsy were independent predictors of any PCa and csPCa in models B.

### Patients’ treatments and ICC

Active surveillance/watchful waiting (20%), surgery (65%), and radiation therapy (10%) were among the treatment options chosen. Additionally, a small percentage of patients underwent focal therapy or other treatments (5%) (Table 2).

ICC between PI-RADS at the first and second mpMRI was 0.43 (0.28–0.54) *p* < 0.01 (Supp Table 3). ICC between PI-RADS at the second MRI and the worst ISUP at re-biopsy was ICC: 0.38 (95%CI: 0.21–0.50) (Supp Table 4). ICCs between the final pathology and RBx biopsy, MRI-TBx, and MRI-TBx + RBx were respectively: 0.53 (0.25–0.70), 0.63 (0.41–0.76), and 0.88 (0.80–0.92) (all *p* values < 0.01) (Supplementary Tables 5, 6, 7).

## Discussion

The present study provides significant findings regarding PCa and csPCa detection in patients with positive mpMRI, negative MRI-TBx plus systematic biopsy, treated with a re-biopsy after a second MRI. Several clinically and radiologically significant changes were observed between the first and second MRI scans and a significant shift in the distribution of PI-RADS scores was observed between the two biopsies. Among the lesions observed in the initial MRI, 42% remained stable, 39% showed upgrading, and 19% exhibited downgrading in the second MRI. Moreover, more than 40% of the patients showed the presence of new lesions at the second MRI. In accordance with our biopsy findings after second MRI, the persistence of the mpMRI positivity should be carefully considered as a trigger for presence of prostate cancer. Other studies have aimed at assessing the medium-term radiological and clinical follow-up of biopsy-negative lesions [11–14]. Specifically, Kornienko et al. [13] found that among 84 men with PI-RADS 4 or 5 lesions who underwent a repeat MRI, more than half of the lesions were downgraded to PI-RADS 3 after a median follow-up of 28 months. However, it is worth noting that 41% of these men, who also underwent repeated biopsy, were diagnosed with clinically significant disease, all of whom had persistent MRI lesions. Stavrinides et al. [14] reported that in the 58 men who had follow-up MRIs, most scores were downgraded, primarily to Likert 3, and this downward trend continued in subsequent MRIs. A small number of patients maintained a Likert 4 phenotype in their serial imaging, and notably, the two men subsequently diagnosed with cancer on follow-up MRI-targeted biopsy consistently had high scores (Likert 4) in their sequential MRI scans. In accordance with a recent systematic review [7], our results support the idea that patients with persistent MRI lesions are at a higher risk of disease. However, drawing definitive conclusions in comparison with previous literature is challenging due to various factors that can influence the studied population, including different targeting, MRI acquisition and protocols [15], reporting protocols (PI-RADS v2 instead of Likert), overall number of included patients, time span between the first and second prostate biopsy, and per protocol defined time between the first and second MRI. Furthermore, it must be notice that in our study we identified low concordance between the subsequent MRIs, underscoring the importance of integrating in the assessment of PI-RADS score, the comparison with previous examinations. This is crucial due to the potential changes that can occur in the prostate over time. The relevance of this approach has already been established with the PRECISE score [16] for patients who are under active surveillance. We strongly believe that a similar approach can also be valuable for specific patient populations, like the one included in the present study.

Indeed, this specific population with high PI-RADS scores and negative target biopsies may reveal some degree of prostate inflammation, which can pose challenges in MRI interpretation. Pepe and Pennisi [17] estimated that approximately 37% of PI-RADS 5 lesions were associated with inflammation. For this reason, it is crucial to determine the histopathology of target biopsy-negative lesions. In our study, we found that the presence of ASAP on the initial biopsy strongly predicted the presence of PCa and csPCa. This supports the growing interest in exploring glandular-stromal alterations, as well as acute or chronic inflammation and vascular changes, which have been observed in a majority of false-positive MRI lesions. Interestingly, these changes are more prevalent and synchronous in MRI-TB tissue compared to systematic biopsy cores from the same patients [18, 19].

In 37.2% of patients, PCa was detected, while csPCa was detected in 25.5% of patients. This highlights the potential limitations of the first TBx and emphasizes the need for further confirmatory biopsies in cases with inconsistent results between MRI images and MRI-guided biopsy findings. Interestingly, the second biopsy involved an increased proportion of software fusion biopsies and targeted transperineal biopsies.

The transperineal approach has a well-established accuracy in cancer detection compared to the transrectal approach [15, 20]. Its role in the re-biopsy is also highlight in the PICTURE study [21]. Transperineal template prostate mapping identified csPCa in 6.5% (11/168; 95% CI 3.3–11%) (Gleason ≥ 3 + 4 of any length or MCCL ≥ 4 mm of any grade) and PCa in 9.3% (20/215; 95% CI 5.8–14%) after a non-MRI-guided transrectal ultrasonography-guided biopsy. Contrary to expectations, the advantages of using targeted transperineal biopsies in our specific patient group were not confirmed [20, 22].

Similarly, the use of MRI-TBx did not significantly enhance the detection abilities of PCa compared to cognitive biopsy. On the contrary, the number of RBx cores was independently associated with the presence of PCa and csPCa, again highlighting the need to combine TBx and RBx. This is also seen in the assessment of the concordance with the final pathology where the combination of systematic and target biopsies was higher. Again, systematic biopsies still play a role in the diagnostic process [23]. These findings are supported by studies where RBx demonstrate greater added value in lobes where an MRI-visible lesion is present compared to those without [24]. This indicates that the primary role of RBx is to detect cancers that are correctly identified on MRI but missed during targeted biopsies or to detect lesions, which were not completely identified by the mpMRI. Additionally, perilesional biopsies appear to enhance the detection rate of csPCa when combined with targeted biopsies alone [25]. Therefore, systematic biopsies can be considered a valuable safety net that compensates for potential quality issues throughout the diagnostic process. It is important to emphasize that the MRI pathway for detecting targeted MRI-visible tumors necessitates diagnosing and treating a significant number of men in order to prevent a single prostate cancer-related death [26]. The inclusion of additional MRIs and biopsies within this pathway may further increase the number of men diagnosed and treated per death prevented. Thus, the more we learn about follow-up procedures, the less stringent we need to be in the initial diagnostic approach.

Our study has several limitations that should be considered. The retrospective design of the study introduces inherent risks of selection bias and no fixed protocol for follow-up. The actual impact of the follow-up regimen, which includes an initial negative biopsy followed by a second MRI and subsequent biopsy, should be compared to other groups that may follow different protocols, such as biopsy without a second MRI or clinical follow-up alone. There were multiple biopsy options available for comparison, including different techniques, software for fusion biopsies, and the number of targeted biopsies. This variability in biopsy options may introduce confounding factors and limit the ability to draw definitive conclusions. Fourthly central readings of imaging were not performed, which could have provided more standardized and reliable results. Due to this limitation, it was not feasible to evaluate the correlation between the PRECISE score of the second MRI and the detection of csPCa. Additional information in the PRECISE score, such as changes in lesion appearance observed in DWI, has the potential to improve the MRI outcomes beyond the PI-RADS score.

Radiologists may exhibit enhanced performance at second MRI, thereby improving their capabilities.

Current evidence indicates that transperineal sampling exhibits improved cancer detection rates for lesions in the anterior/apical region [22]. However, the current study lacks available data to determine whether the access route served as a significant predictor in patients with anterior/apical lesions who initially underwent transrectal biopsy.

Lastly, this study was conducted at multiple centers, introducing the possibility of institutional or regional biases. The variation in practices and patient populations across different centers may influence the study outcomes.

However, these limitations could also be viewed as a strength of this study, as they reflect real-life clinical practice and underscore the significance of follow-up for suspicious MRI findings, even after initial MRI-guided negative biopsies. The implementation of a structured MRI follow-up algorithm, especially for patients with inconclusive results and high-grade PI-RADS scores, can improve diagnostic accuracy and optimize patient care.

## Conclusion

This study highlights the significance of the follow-up needed in the lesion characterization of suspicious MRI findings, even after an initial negative MRI TBx and RBx. The identified of PCa, and above all, csPCa in this setting of patient offers valuable insights for risk assessment and inform decision-making regarding subsequent diagnostic procedures and treatment choices. Patients presenting with inconsistent findings between MRI and prostate biopsy, presence of high-grade PI-RADS, low number of systematic biopsies at first biopsy and presence of ASAP, should undergo thorough evaluation and be included in a structured algorithm for MRI follow-up and eventually repeated biopsy.

This approach is crucial to ensure accurate diagnosis and appropriate management for these individuals, although the long-term prognostic impact of this approach is clearly unknown. Further validation and prospective studies are warranted to confirm and extend these findings to larger patient populations with diverse follow-up regimens and available follow-up protocols. Knowledge on follow-up may relax the initial diagnostic algorithm.

### Supplementary Information

Below is the link to the electronic supplementary material.

Supplementary file1 (DOCX 31 KB)

## Data Availability

Not applicable.
