# Supplementary material for: The impact of a second MRI and re-biopsy in patients with initial negative mpMRI-targeted and systematic biopsy for PIRADS ≥ 3 lesions
Source: World J Urol. 2023 Sep 27;41(11):3357–66. doi: 10.1007/s00345-023-04578-7 (PMC10632220; doi:10.1007/s00345-023-04578-7)
Supplement: Supplementary file 1 — Supplementary file1 (DOCX 31 KB) [file 345_2023_4578_MOESM1_ESM.docx]

**Supplementary table 1.** Clinical and radiological triggers for repeat biopsy.

| MRI upgrading | 17,2% |
| --- | --- |
| MRI lesions number increase | 4,6% |
| MRI lesion diameter/volume increase | 5,7% |
| PI_RADS increase | 1,7% |
| PSA increase | 49,4% |
| DRE increase | 5,7% |
| Other causes | 6,3% |
| Combination | 9,2% |

**Supplementary table 2.** Prostate cancer detection for lesion visualized already at first MRI or New Lesion Detected at Second MRI.

|  | **Negative biopsy** | **Positive Target biopsy** | **Positive Systematic biopsy** | **Positive Systematic +Target biopsy** |
| --- | --- | --- | --- | --- |
| **Lesion visible at first MRI (168 pts)** | **105/168 (62.5%)** | **17/168 (10.1%)** | **19/168 (11.3%)** | **27/168 (16.1%)** |
| **New lesion detected at second MRI (122 pts)** | **77/122 (63.1%)** | **14/122 (11.5%)** | **10/122 (8.2%)** | **21/122 (17.2%)** |

**Supplementary table 3. Intraclass correlation between PI – RADS at first and second MRI**

|  |  | PI – RADS second MRI | | | | |
| --- | --- | --- | --- | --- | --- | --- |
|  |  | Negative MRI | PI – RADS **2** | PI – RADS **3** | PI – RADS **4** | PI – RADS **5** |
| PI – RADS first MRI | PI – RADS 3 | 15 | 5 | 69 | 61 | 9 |
|  | PI – RADS 4 | 4 | 1 | 22 | 72 | 20 |
|  | PI – RADS 5 | 0 | 0 | 0 | 4 | 8 |

ICC 0.43 (0.28 – 0.54) p<0.01

**Supplementary table 4. Intraclass correlation between PI – RADS at second MRI and ISUP at biopsy B**

|  |  | ISUP at biopsy B | | | | | |
| --- | --- | --- | --- | --- | --- | --- | --- |
|  |  | No cancer | ISUP 1 | ISUP 2 | ISUP 3 | ISUP 4 | ISUP 5 |
| PI – RADS second MRI | Negative MRI | 16 | 2 | 0 | 1 | 0 | 0 |
|  | PI – RADS 2 | 6 | 0 | 0 | 0 | 0 | 0 |
|  | PI – RADS 3 | 67 | 7 | 7 | 8 | 1 | 1 |
|  | PI – RADS 4 | 82 | 16 | 22 | 11 | 4 | 2 |
|  | PI – RADS 5 | 11 | 9 | 8 | 7 | 1 | 1 |

ICC: 0.38 (95%CI: 0.21 – 0.50)

**Supplementary table 5. Intraclass correlation between the final pathology for Systematic biopsy**

|  |  | Radical prostatectomy | | | | |
| --- | --- | --- | --- | --- | --- | --- |
|  |  | ISUP 1 | ISUP 2 | ISUP 3 | ISUP 4 | ISUP 5 |
| Systematic biopsy result | Negative | 1 | 5 | 3 | 0 | 1 |
|  | ISUP 1 | 5 | 5 | 1 | 0 | 0 |
|  | ISUP 2 | 0 | 11 | 14 | 1 | 0 |
|  | ISUP 3 | 0 | 1 | 8 | 6 | 0 |
|  | ISUP 4 | 0 | 0 | 0 | 1 | 0 |
|  | ISUP 5 | 0 | 0 | 0 | 0 | 1 |
|  | Not Performed | 0 | 2 | 4 | 2 | 2 |

ICC 0.53 (0.25 – 0.70), p<0.01

**Supplementary table 6. Intraclass correlation between the final pathology for Target biopsy**

|  |  | Radical prostatectomy | | | | |
| --- | --- | --- | --- | --- | --- | --- |
|  |  | ISUP 1 | ISUP 2 | ISUP 3 | ISUP 4 | ISUP 5 |
| Target biopsy result | **Negative MRI** | 2* | 6* | 8* | 1* | 0 |
|  | ISUP 1 | 4 | 7 | 0 | 0 | 0 |
|  | ISUP 2 | 0 | 10 | 9 | 0 | 1 |
|  | ISUP 3 | 0 | 0 | 12 | 4 | 0 |
|  | ISUP 4 | 0 | 0 | 0 | 5 | 0 |
|  | ISUP 5 | 0 | 0 | 0 | 0 | 3 |

ICC 0.63 (0.41 – 0.76), p<0.01

- Target biopsy on the basis of first MRI

**Supplementary table 7. Intraclass correlation between the final pathology for Target and Systematic biopsy**

|  |  | Path ISUP at radical prostatectomy | | | | |
| --- | --- | --- | --- | --- | --- | --- |
|  |  | ISUP 1 | ISUP 2 | ISUP 3 | ISUP 4 | ISUP 5 |
| ISUP at systematic + target biopsies | **Negative MRI** | 0 | 1* | 1* | 0 | 0 |
|  | ISUP 1 | 6 | 7 | 0 | 0 | 0 |
|  | ISUP 2 | 0 | 15 | 10 | 0 | 1 |
|  | ISUP 3 | 0 | 1 | 19 | 5 | 0 |
|  | ISUP 4 | 0 | 0 | 0 | 5 | 0 |
|  | ISUP 5 | 0 | 0 | 0 | 0 | 3 |

ICC 0.88 (0.80 – 0.92) <0.01

* Patients with diagnosis of PCa during follow – up
